# Gunshot Wounds of the Abdomen

**Published:** 1898-07-23

**Authors:** 


### GUNSHOT WOUNDS OF THE ABDOMEN.

Commenting on a case of gunshot wound of the abdomen, in which he had performed immediate laparotomy, with suture of the wounded stomach, and in which the patient had recovered, Mr. Ward Cousins[1] makes some valuable remarks concerning the modern position of surgery in regard to such injuries. He says that we may now accept it as an axiom that in every case of penetrating wound, in which there is reason to believe that an internal injury has been inflicted, the abdomen ought to be opened, with the intention of arresting hæmorrhage, securing by suture any lacerated or wounded organ, removing blood or other extravasated fluids, and cleansing the peritoneal cavity. In cases of gunshot injury, when urgent symptoms are present, it is useless to hope that the viscera have escaped damage, so that as soon as the patient has sufficiently rallied from the shock an exploratory operation is clearly indicated. To wait for more urgent symptoms is to wait for septic poisoning and fatal peritonitis. In many cases the chance of success wholly rests on immediate action. The flickering pulse, the persistent vomiting,

[1] Brit. Med. Jour., July 16.

the cold extremities, are but the outward expression of internal hæmorrhage and extravasation, and the hope of rescue from these conditions hangs on at once exposing the wounded organs and securing them by suture. So far Mr. Ward Cousins is clear and definite, and he expresses with admirable terseness the position of affairs and the only mode of escape; we venture then to express our regret at the limitations which he places upon operative surgery in the following sentence: "It is, of course, useless to operate when the patient is rapidly sinking, for the proper treatment under these circumstances would be hypodermic and rectal stimulation and external heat; and we may rest assured that whenever the judicious application of these remedies has failed to rally the patient, abdominal section would certainly have been of no avail. Even in extreme cases we must not readily relinquish our efforts, for a patient may rally very unexpectedly from a state of profound collapse." This seems to us to reintroduce the question on which all doubt in such cases hinges. It is a matter which, of course, has to be decided by experience and observation; but where there is time for rectal stimulation and external heat to do good, there should be time for the mischief to be got at and perhaps stopped by operation. The matter is one of very great importance, and we hope that Mr. Ward Cousins will reconsider the question of the sinking patient. Even in presence of the profoundest shock a clean incision is not a serious matter, while a continuing extravasation means death.

